# Enhancing Gluten-Free Muffins with Milk Thistle Seed Proteins: Evaluation of Physicochemical, Rheological, Textural, and Sensory Characteristics

**DOI:** 10.3390/foods13162542

**Published:** 2024-08-15

**Authors:** Muhammed Ozgolet, Muhammed Zahid Kasapoglu, Esra Avcı, Salih Karasu

**Affiliations:** 1 Department of Food Engineering, Faculty of Chemical and Metallurgical Engineering, Yildiz Technical University, Davutpasa Campus, Esenler, Istanbul 34210, Turkey; muhammed.ozgolet@gmail.com (M.O.); skarasu@yildiz.edu.tr (S.K.); 2 Department of Nanotechnology, Institute of Nanotechnology and Biotechnology, Istanbul University-Cerrahpaşa, Avcılar, Istanbul 34320, Turkey; 3 Istanbul Teknokent, Istanbul University-Cerrahpaşa, Teknokent Building, Avcılar, Istanbul 34320, Turkey; esravci93@hotmail.com

**Keywords:** milk thistle seed, protein isolate, oilseed by-product, oxidative stability, rheology, gluten-free muffin

## Abstract

This study investigated the potential utilization of milk thistle seed protein (MTP) isolates in gluten-free muffins to enhance the protein quantity and technological attributes. MTP was employed to partially substitute a blend including equal amounts of rice flour and corn starch (RCS) at 3%, 6%, 9%, and 12%. The study encompassed a rheological assessment of muffin batters and physicochemical, textural, and sensory analyses of the muffins. The consistency coefficient (K) of muffin batters exhibited an increase with the incorporation of MTP, with all batters demonstrating shear-thinning behavior (n < 1). The dough samples exhibited solid-like characteristics attributed to G′ > G″, indicative of their viscoelastic nature. The storage modulus (G′) and loss modulus (G″) escalated with higher levels of MTP, suggesting an overall enhancement in dough viscoelasticity. The muffin containing wheat flour displayed the lowest hardness value, followed by MTP-added muffins at ratios of 12% and 9%. Additionally, MTP-added muffins exhibited greater springiness values than control samples without MTP (C2). However, the oxidative stability of MTP-added muffins was lower than the wheat control muffin (C1) and gluten-free control muffin. The protein content in muffins increased with MTP addition, reaching parity with wheat flour muffins at 6% MTP replacement. Sensory analysis revealed that substituting RCS with up to 6% MTP did not significantly alter the overall quality (*p* > 0.05), whereas higher MTP levels (9% and 12%) led to a decline in sensory attributes. Incorporating MTP at up to 6% yielded protein-enriched muffins with sensory characteristics comparable to the wheat flour muffin (C1). Furthermore, higher MTP additions (9% and 12%) conferred more favorable textural properties than the C2 muffin. However, the oxidative stability of the control muffins was found to be higher than that of MTP-added muffins. This study suggested that MTP could be a potential ingredient to increase the protein amount and specific volume of gluten-free muffins and to improve textural attributes such as springiness and hardness.

## 1. Introduction

In recent times, food technologists have shown a keen interest in developing functional and enriched food products with high nutritional value due to increasing consumer awareness of health benefits and their growing acceptance of innovative food offerings. Furthermore, there is a notable trend toward sustainability in the food industry, emphasizing environmental and economic considerations. This has prompted a reevaluation of waste and by-products from food processing as viable raw materials. Notably, by-products derived from oil processing hold significant potential as valuable raw materials due to their rich contents of protein and fiber. Oil cakes and meals, traditionally considered by-products, are now being utilized to produce protein isolates and hydrolysates, as highlighted in recent studies [1,2]. Globally, there are approximately 200 oil-rich plant species [3]. While soybean, rapeseed, sunflower, coconut, and olive are the most prevalent sources of oils, other seeds, like milk thistle, chia, nigella, pumpkin, and hemp, have also been used recently to produce oils. Despite their various chemical compositions, all oilseeds contain high amounts of fiber, vitamins, minerals, fat, and proteins. Oilseeds provide vital protein in amounts ranging from 6% to 45%, and they also contain at least 15% fat [4]. Various oil cakes lead to regulation of the cardiovascular and immune systems [3] and improvements in bone health [5] and brain function [6]. Increased awareness of the health benefits associated with oilseeds and their oils has driven the global expansion of oil plant cultivation [3]. Edible oil cakes, predominantly used as livestock feed due to their high protein content and other bioactive compounds, are now facing a demand for more efficient utilization in light of the growing human population [7]. Consequently, oilseeds and oils are being increasingly integrated into food products, contributing to the expanding global production of oil plants [8].

The increasing demand for gluten-free products has spurred significant expansion within the market segment. Approximately 1% of the global population suffers from celiac disease, and about 6% experience non-celiac gluten sensitivity (NCGS) [9]. Despite these relatively small percentages, the gluten-free product market is projected to grow by 9.2% from 2020 to 2027, driven primarily by the bakery sector [10]. Additionally, there is a growing trend among health-conscious individuals toward gluten-free products. Strict lifetime avoidance of gluten-containing grains, such as wheat, rye, barley, oats, and triticale, is the main treatment for celiac disease and NCGS. The need for these products has raised interest in the creation of gluten-free (GF) foods. However, GF products often lack adequate nutrients and are high in carbohydrates, potentially leading to nutritional deficiencies in the diet [11]. Rice flour is the most common flour used in commercial gluten-free products. Hager et al. [12] compared wheat flour and common gluten-free flour. In this study, wheat flour had higher amounts of protein, fiber, and minerals and a lower amount of phytate than rice flour. Consequently, various studies have explored enriching gluten-free products with proteins, dietary fiber, and other essential nutrients, aiming to enhance their nutritional value, sensory qualities, and consumer acceptance [13,14]. Plant-based proteins are particularly promising for enhancing gluten-free product development due to their ability to improve the nutritional profile, sensory attributes, and dough characteristics such as viscoelasticity [15].

Muffins are widely enjoyed sweet leavened baked goods, commonly consumed as a breakfast or snack. They are prized for their pleasing taste and soft texture. Protein-enriched gluten-free (GF) muffins offer a promising way to enhance the nutritional quality of the diets of individuals with gluten intolerance. Protein-rich oil cakes and protein isolates derived from oil cakes have the potential to be utilized to improve the nutritional and technological attributes of muffins. In the literature, a limited number of studies are present demonstrating the influences of protein isolates on muffin quality. Matos et al. [15] developed rice-flour-based muffins by incorporating diverse gluten-free protein sources to modify the characteristics of gluten-free muffins. They reported that the addition of soy protein isolates notably enhanced the storage modulus of the batter during development and also influenced the textural attributes of the baked products. Shevkani et al. [16] fortified rice-flour-based muffins with cowpea protein isolates in amounts up to 12%. The incorporation of protein isolates significantly enhanced the functional properties of rice flour in muffin preparation. The extent of the improvement varied depending on the added amount and origin of the proteins. The effects of sesame protein isolate on cakes were evaluated with the addition of the transglutaminase enzyme [17]. The incorporation of soy protein isolate (SPI) and transglutaminase (TG) resulted in elevated batter viscosity, specific volume, and porosity, accompanied by decreased cake density and baking loss. On the basis of the literature research, it is concluded that there have been no studies that used milk thistle seed and its oilseed by-products in food formulations. A previous study demonstrated that milk thistle seed proteins offered high thermal stability due to their high β-sheet concentration and good emulsifying and foaming capacities [18]. This study aims to show the potential usage of a protein isolate derived from cold-pressed milk thistle seed by-products (MTP) in a gluten-free muffin formulation by analyzing the effects of MTP on the rheological characteristics of gluten-free muffin batters and the textural features, oxidative stability, and sensory attributes of gluten-free muffins.

## 2. Materials and Methods

### 2.1. Materials

Milk thistle seed proteins were extracted from cold-pressed milk thistle seed oil by-products (protein: 22.6%; fat: 8.0%; moisture: 6.7%; and ash: 4.8%) ordered from a cold-pressed-oil producer (Tazemiz, Mersin, Türkiye). The origin of the milk thistle is Denizli, Türkiye. Milk thistle seed by-products were obtained by grinding oil-free milk thistle seeds after cold-pressing by a cold press oil machine (NF500, Karaerler, Ankara, Türkiye). Then, it was sifted and turned into the flour form. The muffin ingredients were purchased at a local supermarket in Istanbul, Türkiye. All chemicals were ordered from Sigma-Aldrich (St. Louis, MO, USA). 

### 2.2. Methods

#### 2.2.1. Preparation of Milk Thistle Protein Isolate

Milk thistle seed protein (MTP) was obtained from cold-pressed milk thistle seed oil by-products with the optimal extraction parameters described by Ozgolet et al. [18]. In short, the protein was extracted at an optimal pH (9.5), duration (180 min), and temperature (30 °C) combination. Proteins were obtained by acid precipitation at pH 4. Then, the protein pellet was collected by centrifugation at 12,000× *g* for 15 min at 20 °C. After the protein pellet was freeze-dried, the protein amount of the obtained MTP isolate was 84.9%.

#### 2.2.2. Preparation of Gluten-Free Muffin Samples

Muffins were prepared following the procedure proposed by Goksen and Ekiz et al. [19] with slight modifications. In short, the wheat flour in the control muffin with wheat flour (C1) and the blend of corn starch and rice flour (RCS) in the gluten-free control muffin (C2) were replaced by 3–12% milk thistle seed protein (MTP). The muffin formulations are presented in Supplementary Table S1. First, an egg (50 g) was cracked and mixed thoroughly with a stand mixer (Kitchen Aid, MI, USA), followed by sugar addition (60 g). After 2 min of high-speed (Level 8) mixing, the liquid ingredients (oil: 50 g; milk: 50 g) were added and blended at a medium speed (Level 4) for 2 min. Then, the dry ingredients (corn starch: 50 g; rice flour: 50 g; baking powder: 3.3 g; vanillin: 1.5 g) were added to the mixer and blended at low speed (Level 2) for 2 min more. The gluten-free muffin batters (roughly 50 g) were put into a muffin pan and baked in a preheated electrical oven (FIMAK, Konya, Türkiye) at 180 °C for 30 min.

#### 2.2.3. Rheological Characteristics of Gluten-Free Muffin Batters

Steady-shear, dynamic shear, three-interval thixotropy test (3-ITT), and temperature sweep test rheological characteristics of gluten-free muffin batters using a strain- and temperature-controlled rheometer (MCR 302; Anton Paar, Sydney, NSW, Austria). All rheological analyses except the temperature sweep test were carried out at 25 °C, with a plate diameter of 25 mm and a gap of 1 mm. These values were determined based on a preliminary test. All analyses were carried out by using a parallel plate configuration. For steady-shear rheological characteristics, the shear rate ranged from 0.1 to 100 s^−1^. The sample was positioned between the plates, and the test was conducted. The power-law model was used to describe the relationship between the shear stress and shear rate with nonlinear regression (Equation (1)):

(1) $$\tau =K\gamma^{n}$$

Within Equation (1), *τ* stands for shear stress (Pa), *K* stands for the consistency index (Pa·s^n^), *γ* stands for the shear rate (1/s), and n stands for the flow behavior index.

A strain sweep test was used to determine the linear viscoelastic region (LVR) before the dynamic rheological analysis of the gluten-free muffin batters. At 0.1–10 Hz and 0.1–64 ω angular velocity ranges, the frequency sweep test was conducted in the LVR. The storage modulus (*G*′) and loss modulus (*G*″) were determined by oscillation shearing, and the power-law model and nonlinear regression were used to assess the plot of angular frequency versus *G*′ and *G*″.

(2) $$G^{\prime }=K^{\prime }{\left(\omega \right)}^{n^{\prime }}$$

(3) $$G^{\prime \prime }=K^{\prime \prime }{\left(\omega \right)}^{n^{\prime \prime }}$$

In Equations (2) and (3), *G*′ (Pa) stands for the storage modulus, *G*″ (Pa) stands for the loss modulus, *ω* stands for the angular velocity value (1/s), *K*′ and *K*″ stand for consistency index values, and *n*′ stands for the flow behavior.

The 3-ITT test was performed to assess the ability of muffins to recover their structure after shear deformation. Three intervals simulate the resting period at low shear for 100 s (1st interval) before high-shear deformation for 40 s (2nd interval). After high-shear deformation, the same conditions were provided to estimate the regeneration of muffin batter at a low shear rate for 150 s. The deformation (%) during high shear and recovery (%) of the muffin structure were calculated by using Equations (4) and (5). The regeneration of the muffin structure was modeled by fitting a second-order structural model in the third interval.

(4) $$\mathrm{Dr}\;\left(\%\right)=\frac{G_{i}-G_{0}}{G_{i}}\times 100$$

(5) $$\mathrm{Rec}\;(\%)=\frac{G_{30}}{G_{i}}$$

In Equations (4) and (5), *G*_i_ stands for *G*′ values in the first state of the batters, *G*_0_ represents the *G*′ values immediately after shear rate deformation, and *G*_30_ stands for the *G*′ values of the batter within the first 30 s immediately after the shear rate deformation.

(6) $${\left[\frac{G^{\prime }-G_{e}}{G_{0}-G_{e}}\right]}^{1-n}=(n-1)kt+1$$

In Equation (6), G′ stands for the storage modulus (Pa), *k* stands for the thixotropic rate constant, *G*_0_ stands for an initial storage modulus (Pa) in the third time interval, and *G_e_* is the equilibrium storage modulus (product completely recovers itself) [20]. 

The temperature-dependent properties of the muffin batters were tested by a temperature sweep test. The temperature range of 25 °C to 120 °C was used to analyze the fluctuations in the G′ value of the samples. The temperature was ramped at a constant rate (5 °C/min).

#### 2.2.4. Physicochemical Characteristics of Gluten-Free Muffin Samples

The protein content of muffins was determined with the standard AOAC method, numbered 990.03. The Kjeldahl method was employed using a nitrogen-to-protein conversion factor of 6.25. The water activity (aw) of the muffins was analyzed at 30 °C using a Novasina Lab Master aw meter (Novasina AG, Lachen, Switzerland). The structures of muffin crumbs were evaluated with ImageJ2x version 1.54c software, which examined digital images of the crumbs (NIH, USA, https://imagej.nih.gov/ij/, accessed on 15 June 2024). The analysis involved evaluating the contrast differences between the solid and pore phases in order to interpret the images. To quantify the pore area and overall pore area within the crumb (in square millimeters), the photos were first cropped, then converted to grayscale, and lastly, binarized after the threshold was attained. Estimating the ratio of pore area to the complete analyzed area allowed for the determination of porosity [21].

Using calipers, the height of each muffin was measured from the bottom to the peak point on top. The rapeseed displacement method was used to measure the loaf volumes [22]. The specific volume, represented as mL/g, was calculated as the volume-to-weight ratio. The baking loss was calculated by weighing the muffin dough prior to baking and the finished product an hour after baking.

#### 2.2.5. Textural Properties of Gluten-Free Muffin Samples

Using a TA-TX plus Texture Analyzer (Stable Micro Systems, Surrey, UK), a texture profile analysis (TPA) was carried out according to the proposed method in [22]. Muffins were sliced into 20 mm vertical slices from the crumb. The parameters were as follows: compression to 40%, two compression cycles with a five-second interval, a pre- and post-test speed of 1 mm/s, and a test speed of 3.0 mm/s. For the analysis, a 36 mm cylindrical probe and a 5 g trigger force were employed. The TPA curves yielded the following parameters: resilience, cohesiveness, chewiness, hardness, and springiness. The samples were checked on the 3rd and 7th days of storage, as well as two hours after baking.

#### 2.2.6. Oxidative Stability of Muffin Samples

The oxidative stability of the muffin samples was assessed using the OXITEST Device (Velp Scientifica, Usmate, MB, Italy) in compliance with the protocol described in [23]. The device’s receptacle held a 20 g sample, and the accelerated oxidation test was run at 90 °C and 6 bar of pressure. The sample’s redox stability was measured using the induction period (IP, expressed in hours and minutes).

#### 2.2.7. Sensory Analysis

Samples of gluten-free muffins were analyzed using a quantitative descriptive analysis to ascertain their sensory characteristics. During the quantitative descriptive analysis, a sensory panel was employed. The descriptives used were appearance, taste/flavor, aroma, smell/odor, texture/mouthfeel, sweetness, and overall quality. Fifteen trained panelists from Yildiz Technological University’s Food Engineering Department conducted the sensory evaluation. Descriptive scoring was evaluated using 10 cm unstructured line scales, and the scores were converted into numbers on a 5-point scale using a ruler.

#### 2.2.8. Statistical Analysis

All analysis results were given as corresponding mean values together with the standard deviation. To perform multiple comparison tests, Tukey’s test was employed to ascertain the statistical difference between the means at the 0.05 significance level. The Statistica program (version 12; Statsoft, Tulsa, OK, USA) was utilized to obtain the rheological parameters and coefficients of determination (R^2^) of the models. Microsoft Excel software (version 2016; Microsoft Offfice, Redmond, WA, USA) was used to generate the figures.

## 3. Results and Discussion

### 3.1. Rheological Characteristics of Muffin Batters

**Figure 1:**
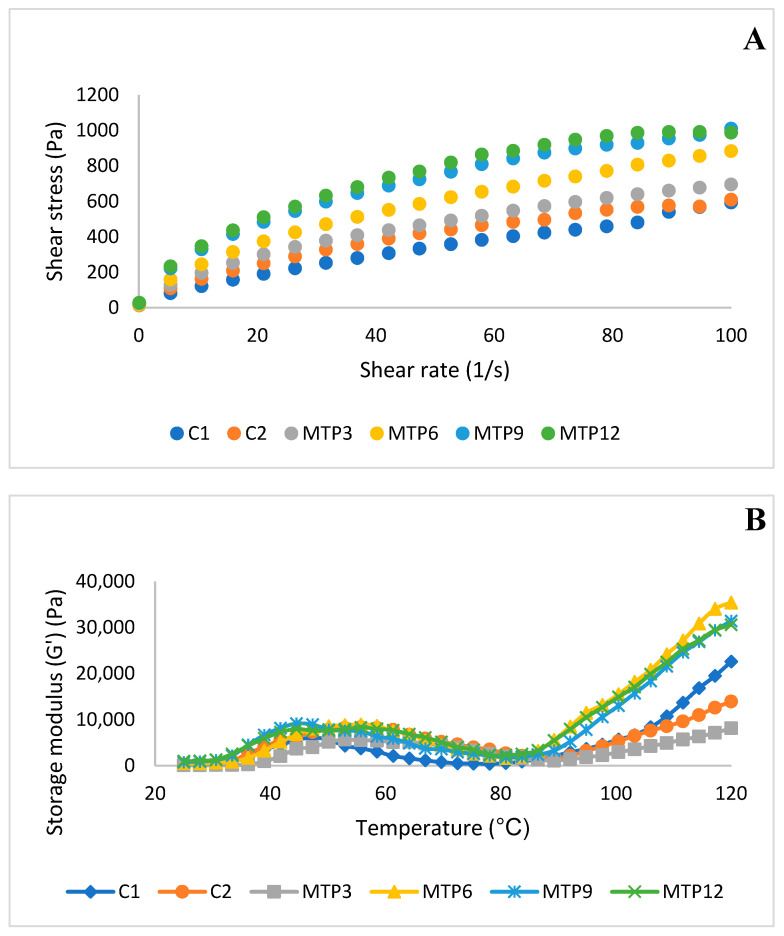
Steady-shear rheological properties and temperature sweep test of muffin batters. Codes: Control formulations with wheat flour (C1) and gluten-free control without MTP (C2), formulations fortified with 3, 6, 9, and 12% milk thistle seed by-product protein isolates (MTP3, MTP6, MTP9, and MTP12, respectively). (**A**): Steady shear, (**B**): temperature sweep test.

Batter consistency is a significant factor since it directly influences gas retention during heating. The optimal muffin batter should be sufficiently dense to prevent air bubbles from escaping during the baking process [13]. The graph of shear stress versus the shear rate of muffin batters is presented in Figure 1A. The shear stress of the batters increased with increasing MTP isolate contents. Figure 1A demonstrates that the viscosity of all samples decreased as the shear rate increased, indicating shear-thinning behavior. The shear-thinning behavior of the gluten-free muffin batter prevents an excessive rise in viscosity, which may cause issues with batter handling, mold filling (metering), machinery cleaning, and/or higher pumping energy costs. In Table 1, the consistency indexes (K) and flow behavior indexes (n) of the muffin batters were obtained using the power-law model. While the K values of muffin batters enriched with MTP varied between 55.58 Pa·s^n^ and 128.15 Pa·s^n^, the K value of the wheat control muffin batter (C1) was 21.00, and the K value of the gluten-free control muffin batter (C2) was 46.46 Pa·s^n^. MTP addition led to an increase in the consistency index (K), which was explained by the synergistic effect of the mixture of muffin ingredients. The flow behavior indexes (n) of MTP muffin batters were found to range between 0.46 and 0.56, which are below those of the C1 muffin. The increase in protein in MTP muffins does not cause a significant change in n values (*p* > 0.05). The fact that the n values of cake doughs are less than 1 indicates that they exhibit non-Newtonian shear-thinning behavior. N was significantly lower, and K was much higher in MTP-added muffin batters than in the control muffin batters, again indicating greater pseudoplasticity and consistency under large deformations [24]. The increase in K value compared to the C1 batter may be associated with the xantham gum in gluten-free muffin formulations. A previous study reported that the addition of xantham gum to the batter formulation increased the viscosity. They also stated that the combined use of soy protein and xantham gum caused higher viscosities in gluten-free muffin batters, which is comparable to our results [25]. The higher consistency of MTP9 and MTP12 may be the main cause of the higher specific volume of these two muffins compared to other muffins.

**Figure 2:**
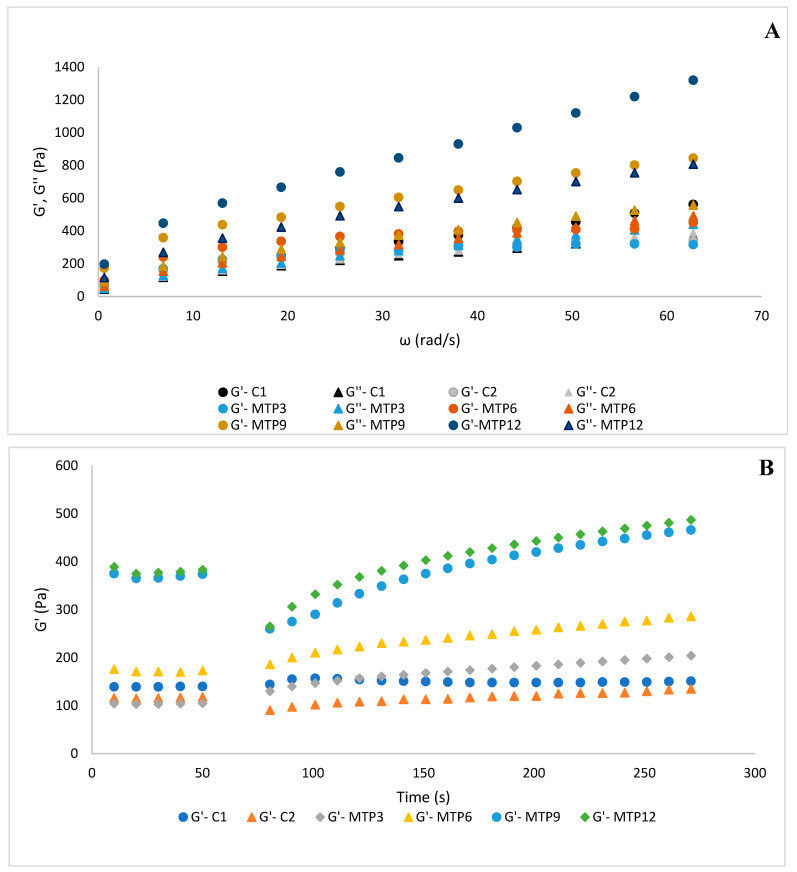
Frequency sweep test (**A**) and 3-ITT rheological properties (**B**) of the muffin batters. Codes: Control formulations with wheat flour (C1) and gluten-free control without MTP (C2). Formulations fortified with 3, 6, 9, and 12% milk thistle seed by-product protein isolates (MTP3, MTP6, MTP9, and MTP12, respectively).

The frequency sweep test was used to describe the dynamic rheological characteristics and viscoelasticity of the muffin batters. The graphs of storage (G′) and loss moduli (G′′) versus frequency are presented in Figure 2A,B. All muffin batters showed soft gel behavior due to their G′ values being higher than G′′. The MTP-added muffin batters had higher G′ and G′′ compared to the C1 and C2 control muffin batters. The more viscoelastic nature of MTP-added batters revealed the presence of a complex structure. A power-law model and nonlinear regression were used to model the changes in G′ and G′′ against angular velocity, and the dynamic flow characteristics of the batters were defined by the obtained K′, K′, n′, and n″ values (Table 1). The K′ values of the muffin doughs with MTP were found to be between 103.53 and 150.66 Pa·s^n^. The K″ values ranged from 38.01 to 99.51 Pa·s^n^, respectively. The fact that the K′ values were higher than K″ in all batters indicates the viscoelastic solid character of the batters. Similarly, the addition of soy and pea protein to the muffin batter altered its viscoelasticity, leading to hardening derived from higher G′ and G″ [11]. Kaur, Singh, and Kaur [26] added different oil cakes to muffin batters, and their addition increased batter viscoelasticity by increasing G′ and G″. The changes were attributed to an increase in the protein amount and water absorption capacity of the flours. G′ showed higher values at all angular velocities, demonstrating the ability of muffin batters to retain air bubbles. In addition, the incorporation of various protein isolates into gluten-free muffin batter increased batter viscoelasticity by increasing G′ and G″. The higher viscoelasticity of protein-added batters was dependent on the ability of the proteins to absorb free water to facilitate the movement of particles in the batter [27]. Lower water activities of MTP-added muffins may cause higher G′ and G″, since the free water facilitates the movement of particles in the batters. 

**Figure 3:**
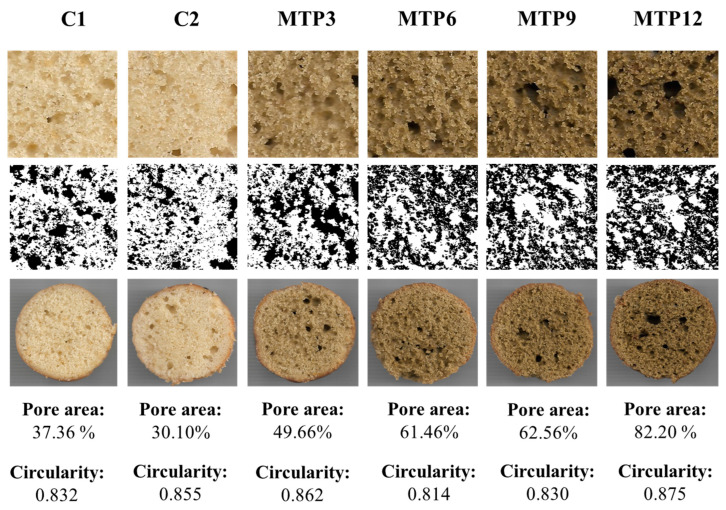
Crumbs of muffin samples visualized by ImageJ2x version 1.54c software. Codes: Control formulations with wheat flour (C1) and gluten-free control without MTP (C2). Formulations fortified with 3, 6, 9, and 12% milk thistle seed by-product protein isolates (MTP3, MTP6, MTP9, and MTP12, respectively).

Figure 3 shows that all batters displayed a thixotropic character in the third interval, indicating that all muffins can regain their viscoelastic character after high-shear-rate deformation. These findings indicated that all samples could maintain their viscoelastic character with a high-shear deformation during food preparation. In the first interval, as expected, MTP addition increased the storage modulus from 87.32 (C2) to 237.05 (MTP12). Second-order model parameters for thixotropic behaviour were presented in Table 2. All batters showed Ge/Go values bigger than 1, indicating the regeneration ability of the batters after high-shear deformation. The G′ values of C2, MTP9, and MTP12 decreased during the second interval, with %Def values of 23.2%, 23.8%, and 30.8%, respectively, showing the loss of their structure. The %Rec values demonstrated that C2, MTP9, and MTP12 were not able to recover their structures in 30 s after high shear, with %Rec lower than 100%. Among these three batters, the time required to reach 100% regeneration was lower in MTP9 and MTP12 compared to C2, indicating that the MTP9 and MTP12 batters recover their structure more readily than C2. In the second interval, there was no structural deformation in C1, MTP3, or MTP6. All batters displayed higher Ge/Go values than C1, demonstrating that the increase in G′ is higher in C2 and MTP-added batters in the third interval. In a previous study, protein addition to a muffin formulation at higher ratios caused considerable deformation compared to the muffin without protein addition [13].

The viscoelastic characteristics of muffin batters during thermal conditions were determined by using a temperature sweep test. The changes in the batters’ storage moduli (G′) during heating from 25 to 120 °C are presented in Figure 1B. The flow behavior of muffin batters depending upon the temperature change gives information about the pasting characteristics. This behavior is mostly dependent on the flour composition and the properties of starches and proteins [16]. G′ increases when starch granules absorb water and swell because of gelatinization during heating, but it then decreases when the starch granules break down at high temperatures. Increasing the temperature above 80 °C led to a rise in both moduli for all muffin batters due to starch gelatinization. The wheat muffin batter showed an onset temperature of gelatinization of around 80 °C. The muffin batters that contained a lower quantity of proteins (C2 and MTP3) showed a higher temperature of starch gelatinization onset. In a previous study, whey and sodium caseinate additions were absorbed by starch granules, thereby decreasing the absorption of water and increasing the gelatinization temperature of starch [28]. Wang et al. [29] also reported that the addition of endogenous protein to rice starch increased the gelatinization temperature. They claimed that this could be associated with the competition of proteins with starch for absorbing water. The MTP-enriched muffins (MTP6, MTP9, and MTP12) showed similar viscoelastic behavior. These batters demonstrated a considerable rise in G′, indicating a more solid and stiff structure when temperature increased. The muffin batters showed a thermal transition between 45 and 55 °C due to protein folding, which resulted in a loss of integrity and a drop in the storage modulus (G′). In the current study, the G′ values increased at the beginning of the temperature increase. Singh et al. [30] and Matos et al. [15] reported similar results, and the increase in G′ at lower temperatures has been attributed to protein–protein interactions [11,25]. After that, the G′ values started to decrease until reaching the gelatinization temperature of starch molecules. This decrease may be due to the denaturation or dissociation of proteins and the disruption of the three-dimensional starch structure [31]. 

### 3.2. Physicochemical Characteristics of Muffin Samples

Table 3 shows the physicochemical characteristics of the muffins. The addition of MTP at higher ratios (MTP9 and MTP12) led to an increase in the specific volume and height of gluten-free muffins. The muffin batters with a high ratio showed higher viscoelasticity. This may cause the formed gases to remain in the muffin batter during baking. The control samples showed lower baking loss values. The changes may be due to an increase in the water absorption capacity of the flours with the addition of proteins. In a previous study, casein, whey protein isolate, soya protein isolate, and egg-white-protein-added muffins had a higher water-holding capacity than gluten-free control muffins, allowing less moisture to escape during baking and, as a result, reducing weight loss [32]. The porosity of MTP-added muffins was higher than that of control samples, and the porosity increased with the addition of MTP. A previous study also reported the addition of protein-rich chia seed oil by-product enrichment caused an increase in the porosity of muffin samples [21]. The circularity values of muffin crumbs ranged between 0.813 and 0.872. A particle’s similarity to a perfect circle is measured by its circularity, which is close to 1 for perfect circles. The higher circularity value was observed in the MTP12 muffin. The circularity values of four different gluten-free muffin crumbs ranged from 0.76 to 0.85, consistent with our results [33]. Pores with a circular shape were found in the muffins, which is characteristic of a well-aerated, evenly distributed muffin core. However, there is no trend between the protein amount and circularity values of the muffin crumbs. The average size of pores and perimeters also increased with increasing MTP amounts, indicating the larger size of pores formed in MTP-added muffins. The protein content in muffins increased with MTP addition, reaching parity with wheat flour muffins at 6% MTP replacement. The water activity of MTP3 was higher than that of control muffins. However, when exceeding 3%, the water activity values of gluten-free muffins decreased.

### 3.3. Textural Properties of Muffin Samples

The TPA parameters of the control and MTP-added muffins were obtained on the first day, third, and seventh day (Table 4). A texture analyzer was used to compress the samples twice during the TPA test in order to gather information about how the samples behave during chewing. The hardness of the muffins varied from 6.70 to 7.98 N on the 1st day. The hardness represents the peak force during the first compression. As expected, during seven days of storage, the hardness of the muffins increased. The C1, MTP9, and MTP12 muffins displayed lower hardness values on the 1st day. The increase rate of hardness was higher in C2 and MTP-added muffins during storage. However, MTP9 and MTP12 showed lower hardness values than the C2 muffin on all days. Shevkani and Singh [27] added kidney bean, pea, and amaranth proteins to a gluten-free muffin formulation (10%). Pea and amaranth proteins caused a decline in hardness from 15.4 N to 7.9 and 11.9 N, respectively. Additionally, MTP-added muffins exhibited greater springiness values than those with wheat (C1) and RCS flour (C2) on the 1st day. Springiness quantifies the ability of a material to recover its original shape after being subjected to deformation during an initial compression. After seven days of storage, all MTP-added muffins except MTP3 showed higher springiness values than the C2 muffin. High springiness values indicate higher-quality muffins. Springiness is linked to fresh, aerated, and elastic texture. This may be attributed to the increased porosity of the gluten-free muffin batters. In addition, rheological studies showed that MTP addition caused an increase in both elastic and viscous behavior, leading to the viscoelastic nature of gluten-free muffin batters. In a previous study, two different protein isolates also showed favorable effects on the springiness value. Beyond 8%, cowpea protein addition led to an increase in the springiness value [16]. The authors associated the results with increased volume and porosity thanks to the increased batter viscoelasticity and surface-active properties of proteins stabilizing the air bubbles in gluten-free muffin batters. In the same study, the cohesiveness values of muffins increased with the addition of 8% and 12% cowpea protein isolate. Similarly, the cohesiveness values of MTP muffins were higher than those of the control muffins on the 1st day. However, during storage, the C1 muffin showed the highest cohesiveness values, followed by MTP12 and MTP9. Cohesiveness measures the muffin structure’s internal resistance to compression. Both the cohesiveness and springiness values of MTP-enriched muffins were higher than those of the C2 muffin, indicating that more elastic and less crumbly muffins can be prepared by MTP addition. The chewiness values of the C1 and MTP9 muffins were lower on the 1st day. C2 and MTP3 demonstrated lower chewiness values, probably due to lower cohesiveness values after 7 days of storage. The resilience values of MTP-added muffins were higher compared to C1 and C2 muffins. Resilience represents the capacity of a material to recover its original height after being compressed or deformed. During storage, the resilience values of MTP muffins decreased faster than the control muffins. The C1 muffin showed the highest resilience value, while the MTP3 muffin showed the lowest resilience value. The other muffins did not differ from each other (*p* > 0.05). The rheological analysis above indicated that the viscoelasticity of muffin batters was enhanced with the addition of MTP, leading to higher springiness and resilience values and more aerated muffins. Consequently, the textural attributes of MTP-added muffins, particularly MTP9 and MTP12, were comparable to those of the C1 muffin on the 1st day. However, during storage, MTP-added muffins were not able to preserve their superior textural features as much as the C1 muffin. The delaying effect of wheat glutenin on starch retrogradation may be the main reason for the preserved textural characteristics of the C1 gluten-free muffin. Kuang et al. [34] stated that the retrogradation of starch may be inhibited by interactions between the gluten network and gelatinized starch. Glutenin primarily interfered with starch’s ability to retrograde by competing with starch for moisture or by preventing starch chains from reassociating. However, in any case, the MTP9 and MTP12 muffins displayed a more desirable texture than the C2 muffin, with lower hardness and higher springiness and resilience values on the 1st day and after 7 days of storage. 

### 3.4. Oxidative Stability of Muffin Samples

The induction periods (IPs) of the control and MTP-added muffins at 90 °C and 6 bar are given in Table 3. The IP of MTP-added muffins (between 8:40 and 9:32 h) was lower than the IP value of the control samples (C1—10:50 h; C2—11:08 h), demonstrating that MTP decreased the oxidative stability of the muffins. Al-Neshawy and Al-Eid [35] showed that wheat gluten enhanced the oxidative stability of vegetable oils. This was attributed to heat treatments denaturing the gluten and releasing sulfur compounds, which have antioxidant properties. This may be the reason for the higher oxidative stability of the C1 muffin. As mentioned before, the addition of protein increased the gelatinization temperature. The gelatinization process increases the accessibility of cereal enzymes to starch, especially when fully gelatinized. This produces glucose, which is more active in reducing sugar, promoting the Maillard reaction (MR). Many studies have shown the antioxidant potential of MR products in model systems and bakery products [36,37]. The delay of the MR due to protein addition may hinder the formation of antioxidative MR products, thereby decreasing oxidative stability in MTP-added muffins. In addition, the higher starch amount in the C2 muffin may lead to a higher rate of starch–lipid complexes, which increase the oxidative and thermal stability of lipids [38].

### 3.5. Sensory Analysis

The sensory characteristics of the muffins, specifically the attributes of appearance, taste/flavor, smell/odor, texture/mouthfeel, sweetness, and overall quality, are presented in Table 5. The addition of a high amount of MTP caused a decrease in appearance values (MTP9 and MTP12). This is probably due to the dark colors of MTP-enriched muffins at high ratios. The taste/flavor values of the C1 muffin were higher compared to other muffins, while the other muffins were not different from each other (*p* > 0.05). Similar results were observed in the texture/mouthfeel sense. These results were consistent with the textural attributes. The integrity of gluten-free muffin crumbs was lower, as shown in TPA. Gluten-free muffins had a less elastic structure that easily crumbled. The panelists gave the highest score to the C1 muffin, and the other muffins did not differ from each other. The sweetness of the muffins was not significantly different (*p* > 0.05). In terms of overall quality, although the C1 muffin had the highest score, it was not different from the C2, MTP3, and MTP6 muffins. However, the MTP9 and MTP12 muffins had lower overall quality scores. The results showed that enrichment with MTP had no unfavorable impact on the sensory attributes of the muffins up to the 6% addition. Higher MTP levels (9% and 12%) led to a decline in sensory attributes. Wendin et al. [39] studied different protein-rich flours to be further analyzed in terms of sensorial features. Their results showed that all proteins had a negative impact on the sensory attributes in one or more ways, demonstrating the complexity of various protein fortifications in a basic muffin recipe. The fortification of the cakes with 30% whey, pea, rice, and egg-white proteins decreased the overall acceptability scores in a sensory analysis [40].

## 4. Conclusions

In conclusion, protein enrichment increased the viscoelastic character of muffin batters, and all batters demonstrated shear-thinning behavior and a solid-like gel structure. The textural attributes of the MTP-added muffins were superior to those of the C2 muffin, indicating that protein addition improved the texture of the gluten-free control muffin. MTP-enriched muffins exhibited a more porous structure and higher specific volume than the C2 muffin. Conversely, the oxidative stability of MTP-added muffins decreased. There were no significant differences in the sensorial evaluation of the MTP3, MTP6, and C2 muffins. However, higher MTP additions (MTP9 and MTP12) caused a decline in sensory attributes. MTP enrichment provided higher-quality muffins in terms of texture, volume, and protein quantity compared to the C2 muffin without compromising any sensorial features of the muffins. The C1 muffin showed significantly higher sensorial attributes and textural properties. When exceeding 6% MTP addition, the sensorial attributes of gluten-free muffins were influenced unfavorably. Conversely, higher percentages of MTP (MTP9 and MTP12) offered better textural properties than MTP3 and MTP6. The results showed that the direct MTP addition to the formulation had limited development of muffins. Exploring the utilization of proteins alongside other macromolecules, either as protein hydrogels or oleogels, in gluten-free muffins and other food items may be a subject for additional research. Therefore, the sensorial attributes and oxidative stability of muffins may be improved by the usage of proteins alone or with other macromolecules in gel forms.

## Figures and Tables

**Table 1:** Power-law parameters defining steady-shear and dynamic rheological properties of muffin batters.

	K	n	R^2^	K′	n′	R^2^	K″	n″	R^2^
C1	21.00 ± 1.15 ^f^	0.72 ± 0.06 ^a^	0.997	130.79 ± 3.75 ^c^	0.61 ± 0.02 ^a^	0.992	39.35 ± 2.01 ^d^	0.54 ± 0.03 ^ab^	0.998
C2	46.46 ± 1.37 ^e^	0.56 ± 0.04 ^b^	0.998	86.52 ± 2.22 ^e^	0.34 ± 0.03 ^cd^	0.996	37.92 ± 2.27 ^d^	0.56 ± 0.03 ^ab^	0.997
MTP3	55.58 ± 1.87 ^d^	0.55 ± 0.02 ^b^	0.999	103.53 ± 3.16 ^d^	0.29 ± 0.02 ^d^	0.985	38.01 ± 2.20 ^d^	0.58 ± 0.02 ^a^	0.997
MTP6	67.79 ± 2.07 ^c^	0.56 ± 0.03 ^b^	0.999	142.47 ± 2.69 ^b^	0.28 ± 0.04 ^d^	0.989	50.03 ± 3.13 ^c^	0.55 ± 0.04 ^ab^	0.996
MTP9	112.79 ± 2.52 ^b^	0.48 ± 0.04 ^b^	0.998	158.44 ± 3.82 ^a^	0.40 ± 0.04 ^c^	0.994	66.67 ± 3.27 ^b^	0.51 ± 0.02 ^ab^	0.994
MTP12	128.15 ± 3.62 ^a^	0.46 ± 0.05 ^b^	0.994	150.66 ± 3.95 ^ab^	0.51 ± 0.04 ^b^	0.992	99.51 ± 2.84 ^a^	0.50 ± 0.03 ^b^	0.997

Codes: Control formulations with wheat flour (C1) and gluten-free control without MTP (C2). Formulations fortified with 3, 6, 9, and 12% milk thistle seed by-product protein isolates (MTP3, MTP6, MTP9, and MTP12, respectively). The different lowercase letters in the same column indicate statistical significance (*p* < 0.05).

**Table 2:** Second-order structural kinetic model parameters for 3-ITT.

Sample	G_0_	G_e_	G_e_/G_0_	*k* × 1000	R^2^	%Def	%Rec	R_time (s)_
C1	143.47 ± 2.95 ^c^	154.35 ± 3.00 ^e^	1.08	6.20 ± 0.20 ^b^	0.995	-	111.4	-
C2	87.32 ± 2.37 ^d^	160.39 ± 1.76 ^e^	1.85	7.17 ± 0.35 ^b^	0.988	23.2	89.8	95.5
MTP3	90.71 ± 2.39 ^d^	251.85 ± 4.40 ^d^	2.74	3.57 ± 0.35 ^c^	0.998	-	144.8	-
MTP6	176.40 ± 6.60 ^b^	359.31 ± 6.36 ^c^	2.03	6.42 ± 0.24 ^b^	0.994	-	125.4	-
MTP9	235.89 ± 3.51 ^a^	631.51 ± 5.56 ^a^	2.69	2.79 ± 0.19 ^c^	0.994	30.5	84.0	69.7
MTP12	237.05 ± 4.75 ^a^	587.77 ± 4.01 ^b^	2.48	11.46 ± 0.65 ^a^	0.998	30.8	91.9	51.5

Codes: Control formulations with wheat flour (C1) and gluten-free control without MTP (C2). Formulations fortified with 3, 6, 9, and 12% milk thistle seed by-product protein isolates (MTP3, MTP6, MTP9, and MTP12, respectively). The different lowercase letters in the same column indicate statistical significance (*p* < 0.05). G_0_: the initial value of the storage modulus; Ge: the equilibrium storage modulus; k: the rate constant of recovery of the sample; R^2^: coefficient of determination.

**Table 3:** Physicochemical characteristics of muffin samples and oxidative stability.

Sample	Specific Volume (cm^3^)	Height (cm)	Baking Loss (%)	Porosity (%)	Circularity	Protein (%)	1st Day	A_w_ 4th Day	7th Day	IP (h:min)
C1	2.02 ± 0.03 ^cd^	45.3 ± 0.2 ^c^	9.74 ± 0.26 ^d^	37.66 ± 1.37 ^d^	0.832 ± 0.005 ^c^	5.39 ± 0.07 ^bc^	0.782 ± 0.003 ^c^	0.754 ± 0.003 ^cd^	0.746 ± 0.002 ^abc^	10:54 ± 0.49 ^a^
C2	1.95 ± 0.03 ^d^	45.2 ± 0.2 ^c^	9.88 ± 0.14 ^cd^	30.39 ± 2.06 ^e^	0.856 ± 0.003 ^b^	3.63 ± 0.06 ^e^	0.788 ± 0.002 ^b^	0.758 ± 0.003 ^bc^	0.753 ± 0.003 ^a^	11:08 ± 0.32 ^a^
MTP3	2.17 ± 0.04 ^a^	45.0 ± 0.2 ^c^	10.14 ± 0.13 ^cd^	50.31 ± 3.11 ^c^	0.861 ± 0.003 ^ab^	4.11 ± 0.07 ^d^	0.794 ± 0.002 ^a^	0.774 ± 0.001 ^a^	0.751 ± 0.002 ^ab^	8:40 ± 0.49 ^b^
MTP6	2.07 ± 0.03 ^bc^	45.4 ± 0.2 ^c^	10.31 ± 0.23 ^bc^	60.73 ± 1.92 ^b^	0.813 ± 0.007 ^d^	5.22 ± 0.11 ^c^	0.789 ± 0.001 ^ab^	0.763 ± 0.003 ^b^	0.738 ± 0.002 ^d^	9:32 ± 0.21 ^b^
MTP9	2.10 ± 0.03 ^ab^	48.3 ± 0.5 ^a^	11.03 ± 0.10 ^a^	62.69 ± 1.35 ^b^	0.830 ± 0.006 ^c^	5.57 ± 0.07 ^b^	0.788 ± 0.002 ^bc^	0.751 ± 0.002 ^d^	0.745 ± 0.004 ^bc^	9:07 ± 0.17 ^b^
MTP12	2.06 ± 0.04 ^bc^	47.0 ± 0.5 ^b^	10.75 ± 0.27 ^ab^	79.88 ± 2.64 ^a^	0.872 ± 0.003 ^a^	6.13 ± 0.10 ^a^	0.784 ± 0.002 ^bc^	0.749 ± 0.003 ^d^	0.741 ± 0.002 ^cd^	9:19 ± 0.19 ^b^

Codes: Control formulations with wheat flour (C1) and gluten-free control without MTP (C2); formulations fortified with 3, 6, 9, and 12% milk thistle seed by-product protein isolates (MTP3, MTP6, MTP9, and MTP12, respectively). IP: Induction period. The different lowercase letters in the same column indicate statistical significance (*p* < 0.05).

**Table 4:** Texture profile analysis of muffins fortified with milk thistle seed protein isolate.

Parameters	Storage Day	C1	C2	MTP3	MTP6	MTP9	MTP12
Hardness (N)	1	6.96 ± 0.18 ^b^	7.81 ± 0.21 ^a^	7.92 ± 0.14 ^a^	7.98 ± 0.09 ^a^	6.70 ± 0.19 ^b^	6.81 ± 0.13 ^b^
	4	13.76 ± 0.13 ^c^	23.58 ± 0.34 ^a^	23.65 ± 0.64 ^a^	23.27 ± 0.47 ^a^	20.45 ± 0.60 ^b^	19.42 ± 0.42 ^b^
	7	19.50 ± 0.38 ^c^	28.65 ± 0.92 ^a^	29.16 ± 1.26 ^a^	30.58 ± 0.51 ^a^	26.44 ± 0.71 ^b^	24.37 ± 0.62 ^b^
Springiness	1	0.914 ± 0.005 ^b^	0.925 ± 0.006 ^b^	0.951 ± 0.009 ^a^	0.953 ± 0.005 ^a^	0.950 ± 0.005 ^a^	0.962 ± 0.004 ^a^
	4	0.888 ± 0.004 ^b^	0.826 ± 0.006 ^d^	0.871 ± 0.010 ^c^	0.869 ± 0.004 ^c^	0.904 ± 0.004 ^a^	0.895 ± 0.004 ^ab^
	7	0.846 ± 0.004 ^b^	0.796 ± 0.004 ^c^	0.783 ± 0.006 ^c^	0.850 ± 0.008 ^b^	0.870 ± 0.011 ^a^	0.841 ± 0.008 ^b^
Cohesiveness	1	0.692 ± 0.003 ^b^	0.664 ± 0.006 ^c^	0.719 ± 0.015 ^a^	0.714 ± 0.007 ^ab^	0.731 ± 0.011 ^a^	0.729 ± 0.005 ^a^
	4	0.553 ± 0.008 ^a^	0.383 ± 0.012 ^e^	0.461 ± 0.010 ^cd^	0.454 ± 0.012 ^d^	0.485 ± 0.005 ^bc^	0.493 ± 0.006 ^b^
	7	0.471 ± 0.009 ^a^	0.327 ± 0.012 ^d^	0.328 ± 0.003 ^d^	0.371 ± 0.009 ^c^	0.377 ± 0.008 ^c^	0.410 ± 0.010 ^b^
Chewiness	1	4.410 ± 0.114 ^c^	4.795 ± 0.056 ^b^	5.407 ± 0.036 ^a^	5.432 ± 0.137 ^a^	4.660 ± 0.180 ^bc^	4.787 ± 0.076 ^b^
	4	6.755 ± 0.180 ^c^	7.454 ± 0.380 ^c^	9.498 ± 0.163 ^a^	9.179 ± 0.102 ^ab^	8.958 ± 0.325 ^ab^	8.572 ± 0.315 ^b^
	7	7.772 ± 0.030 ^c^	7.474 ± 0.536 ^c^	7.493 ± 0.340 ^c^	9.639 ± 0.296 ^a^	8.675 ± 0.309 ^b^	8.303 ± 0.085 ^bc^
Resilience	1	0.337 ± 0.005 ^c^	0.356 ± 0.006 ^b^	0.378 ± 0.010 ^a^	0.374 ± 0.006 ^ab^	0.375 ± 0.005 ^a^	0.377 ± 0.008 ^a^
	4	0.245 ± 0.005 ^a^	0.177 ± 0.007 ^c^	0.228 ± 0.008 ^ab^	0.217 ± 0.005 ^b^	0.231 ± 0.006 ^ab^	0.228 ± 0.007 ^ab^
	7	0.199 ± 0.007 ^a^	0.174 ± 0.004 ^b^	0.157 ± 0.007 ^c^	0.177 ± 0.007 ^b^	0.178 ± 0.003 ^b^	0.185 ± 0.004 ^ab^

Codes: Control formulations with wheat flour (C1) and gluten-free control without MTP (C2); formulations fortified with 3, 6, 9, and 12% milk thistle seed by-product protein isolates (MTP3, MTP6, MTP9, and MTP12, respectively). The different lowercase letters in the same row indicate statistical significance (*p* < 0.05).

**Table 5:** Sensory characteristics of muffins fortified with milk thistle seed protein isolate.

	Appearance	Taste/Flavor	Smell/Odor	Texture/Mouthfeel	Sweetness	Overall Quality
C1	4.69 ± 0.48 ^a^	4.39 ± 0.77 ^a^	4.15 ± 0.69 ^a^	4.46 ± 0.66 ^a^	4.38 ± 0.77 ^a^	4.46 ± 0.66 ^a^
C2	4.38 ± 0.77 ^ab^	4.00 ± 1.00 ^ab^	4.23 ± 0.73 ^a^	3.77 ± 1.09 ^ab^	4.15 ± 0.90 ^a^	4.15 ± 0.80 ^ab^
MTP3	4.23 ± 0.73 ^ab^	3.92 ± 0.76 ^ab^	4.15 ± 0.56 ^a^	3.69 ± 0.63 ^ab^	3.92 ± 0.86 ^a^	3.85 ± 0.69 ^ab^
MTP6	4.08 ± 0.76 ^ab^	3.77 ± 0.60 ^ab^	4.08 ± 0.64 ^a^	3.69 ± 0.63 ^ab^	4.00 ± 0.91 ^a^	3.92 ± 0.64 ^ab^
MTP9	3.85 ± 0.69 ^b^	3.31 ± 0.95 ^b^	3.92 ± 0.76 ^a^	3.62 ± 0.51 ^ab^	3.69 ± 1.11 ^a^	3.39 ± 0.65 ^b^
MTP12	3.77 ± 0.73 ^b^	3.31 ± 1.03 ^b^	3.77 ± 0.60 ^a^	3.54 ± 0.78 ^b^	3.46 ± 1.20 ^a^	3.46 ± 0.78 ^b^

Codes: Control formulations with wheat flour (C1) and gluten-free control without MTP (C2); formulations fortified with 3, 6, 9, and 12% milk thistle seed by-product protein isolates (MTP3, MTP6, MTP9, and MTP12, respectively). The different lowercase letters in the same column indicate statistical significance (*p* < 0.05).

## Data Availability

The original contributions presented in the study are included in the article/Supplementary Materials; further inquiries can be directed to the corresponding author.

## Supplementary Materials

The following supporting information can be downloaded at https://www.mdpi.com/article/10.3390/foods13162542/s1: Supplementary Table S1: The formulations of control and protein-enriched muffins with MTP.
