# Supplementary material for: Enhancing Gluten-Free Muffins with Milk Thistle Seed Proteins: Evaluation of Physicochemical, Rheological, Textural, and Sensory Characteristics
Source: Foods. 2024 Aug 15;13(16):2542. doi: 10.3390/foods13162542 (PMC11353771; doi:10.3390/foods13162542)
Supplement: Supplementary file 1 [file foods-13-02542-s001.zip › foods-3134979-supplementary.pdf]

**Table S1.** The formulations of control and protein-enriched muffins with MTP

| Ingredients | C1 | C2 | MTP3 | MTP6 | MTP9 | MTP12 |
|---|---|---|---|---|---|---|
| **Rice flour (g)** | 0 | 50 | 48.5 | 47 | 45.5 | 44 |
| **Corn starch (g)** | 0 | 50 | 48.5 | 47 | 45.5 | 44 |
| **Sugar (g)** | 60 | 60 | 60 | 60 | 60 | 60 |
| **Egg (g)** | 50 | 50 | 50 | 50 | 50 | 50 |
| **Milk (g)** | 50 | 50 | 50 | 50 | 50 | 50 |
| **Sunflower oil (g)** | 50 | 50 | 50 | 50 | 50 | 50 |
| **Baking powder (g)** | 3.3 | 3.3 | 3.3 | 3.3 | 3.3 | 3.3 |
| **Xantham gum (g)** | 0 | 0.1 | 0.1 | 0.1 | 0.1 | 0.1 |
| **Protein (g)** | 0 | 0 | 3 | 6 | 9 | 12 |
| **Vanilin (g)** | 1.5 | 1.5 | 1.5 | 1.5 | 1.5 | 1.5 |
| **Wheat flour (g)** | 100 | 0 | 0 | 0 | 0 | 0 |
